# Lancaster Medical Institute

**Published:** 1840-10

**Authors:** 


### THE WESTERN JOURNAL.

Vol. II.—No. X.

LOUISVILLE, OCTOBER 1, 1840.

### LANCASTER MEDICAL INSTITUTE.

We regard every association for professional improvement, as entitled to commendation. Under this feeling, we have already noticed two or three in the sister States of Kentucky and Ohio; and propose to make known to our readers, all with which we may happen to become acquainted. On a late visit to Lancaster, in the latter State, we had the pleasure of learning something of a society, composed of its respectable physicians, which is manifestly exerting a beneficial influence on the character of the profession in that place. We have prefixed its title to this article. Its President, is Dr. James White, Vice President, Dr. G. W. Boerstler, Secretary, Dr. M. Z. Kreider, and Treasurer, Dr. H. H. Waite.

The primary object of this Institute, is "the advancement of medical science *among its members*." To this end, it holds semi-monthly meetings in November, December, January, February and March, and monthly meetings, during the remainder of the year. At each meeting, a topic is selected for discussion at the next. It is expected that all interesting clinical cases will be reported to the Institute, and the members are furthermore invited to read essays, on the subjects they may investigate, either in medicine or the auxiliary sciences. At the end of his annual term of service it is the duty of the President to deliver a scientific discourse. All these productions are subjected to discussion. It is a further object to regulate the intercourse, and promote the harmony of its members: also to subscribe for Medical Journals.

Now, if the physicians of every country town in the Valley of the Mississippi, were thus to unite, it would unquestionably give an immediate impulse to the profession. They would all be made abler, happier, and more respectable. And why should they not thus unite? Why not resolve to apply their shoulders to the wheels of their profession, and in a spirit of noble emulation, give it a long push, a strong push and a push all together? This would certainly be preferable to the push-pin employments, in which so many of us push away the hours not devoted to the sick; and although, at first the new custom might make us feel a little awkward, it would soon grow into a habit, of which no one would be ashamed. D.

